# Molecular Adhesion Between Asphalt and Glass Fiber-Reinforced Composites from Recycled Wind Turbine Blades in Dry and Hydrated Conditions

**DOI:** 10.3390/ma18173936

**Published:** 2025-08-22

**Authors:** Jiehao Feng, Shuliang Wang, Fan He, Chuanhai Wu, Zhixiang Wang, Fen Du, Dryver Huston, Mandar Dewoolkar, Ting Tan

**Affiliations:** 1 School of Civil Engineering, Sun Yat-Sen University, Zhuhai 519000, China; fengjh36@mail2.sysu.edu.cn (J.F.); wangshliang@mail2.sysu.edu.cn (S.W.); 15989123788@163.com (F.H.); 2 Guangdong Key Laboratory of Oceanic Civil Engineering, Zhuhai 519000, China; 3 Zhuhai Planning and Design Research Institute, Zhuhai 519088, China; 4 Guangdong Hualu Transport Technology Co., Ltd., No. 1180 Guangcong Eighth Road, Guangzhou 510435, China; chwu1972@126.com (C.W.); zxwang2012@yeah.net (Z.W.); 5 School of Industrial Automation, Beijing Institute of Technology, Zhuhai 519088, China; fendu@bit.edu.cn; 6 Department of Mechanical Engineering, University of Vermont, Burlington, VT 05405, USA; dhuston@uvm.edu; 7 Department of Civil and Environmental Engineering, University of Vermont, Burlington, VT 05405, USA; mdewoolk@uvm.edu

**Keywords:** glass fiber-reinforced composites, adhesion, asphalt, aggregates, hydrated

## Abstract

A large number of wind turbine blades will be retired in the near future. Glass fiber-reinforced composites from retired blades, due to their extraordinary strength, toughness, and durability, are promising aggregate candidates in asphalt mixtures. This work studied the interfacial behavior between asphalt and glass fiber-reinforced composites through combined molecular modeling and experimental approaches. Predictions from molecular modeling were first verified through experimental findings using particle probe scanning force microscopy. Then, molecular simulations were conducted to examine the chemical adhesion between binders and aggregates made from minerals and wind turbine blades. The results showed that epoxy–binder adhesion was higher than calcite–binder and silica–binder adhesion but lower than alumina–binder adhesion, denoting that the glass fiber composite aggregates were comparable in chemical adhesion to mineral aggregates. The adhesion was primarily due to van der Waals forces (>80%). Furthermore, the dependence of epoxy–asphalt adhesion on loading rates was examined, during which the high-speed, transitions, and low-speed regions were identified. The impact of water on interfacial behavior was illustrated by examining how water molecules infiltrated interfaces between aggregates and binders at different speeds. The results showed that interfacial adhesion in a hydrated state at low speeds was 20–40% lower than that in a dry state, whereas at high speeds, interfacial adhesion in a hydrated state was 5–15% higher than that in dry conditions. These results could provide essential guidance for the application of retired wind turbine blades as asphalt aggregates.

## 1. Introduction

Wind energy has played a prominent role in supplying energy all over the world, with offshore wind turbines being widely used [1,2,3,4]. The installed wind energy reached 1021 GW in 2023. To improve the efficiency of wind harnessing, the sizes of wind turbine blades have exceeded 100 m [5]. For example, the length of recent offshore wind turbine blades was 117 m [6]. The design lifespan of wind turbine blades is about 20 years [7]. Thus, wind turbines installed in the early 2000s will be retired in the next few years, generating large amounts of retired wind turbine blades. Similar situations are expected for wind turbine blades in other countries. It is estimated that by 2050, about 43.4 million tons of wind turbine blades will be retired across the world [8].

Wind turbine blades are manufactured from fiber-reinforced polymeric (FRP) composites, consisting of continuous fibers and matrices [9]. These composites are engineered to serve in harsh marine environments [10,11]. The fiber reinforcements are mainly glass fibers or hybrid glass and carbon fibers, whereas different epoxies have been used as matrices [12]. The resulting fiber-reinforced composites are light, strong, and durable, and were mainly designed and manufactured to resist significant fatigue loads during the service period of the wind turbine blades. However, these features also make the utilization of retired wind turbine blades a serious challenge, especially with the large number of retired wind turbine blades anticipated in the coming years [13,14]. Currently, several approaches have been employed to deal with retired wind turbine blades, such as incineration, landfill, cement kiln, pyrolysis, resin recycling, and composite applications. There are advantages and disadvantages to these approaches. Although incineration and landfill disposal have been implemented, they are not favored due to environmental concerns [15]. Cement kilning uses glass fiber-reinforced polymer (GFRP) composites as a source to produce cement, but the melting of glass fibers possesses the potential for explosion [16]. The pyrolysis of GFRP generates oil products that are neither efficient nor eco-friendly to use [17]. Recycled resin has been attracting interest throughout the world, but the method lacks fiber reuse [18]. Wind turbine blades have also been shaped into different structural components, such as benches in parks. In addition, GFRP composites from retired wind turbine blades have been manufactured into powders or fibers to reinforce cementitious composites, during which the fabrication process consumes significant energy due to the small sizes of the particles or fibers.

Studies have been conducted on GFRP fiber-reinforced cementitious composites [19,20,21,22,23,24]. Baturkin et al. [25,26,27,28] created FRP needles using GFRP rebars and recycled wind turbine blades, with a nominal diameter of 6 mm and a length of 100 mm (aspect ratio of 17) to replace 5–10% natural aggregates in concrete. Research [25,26,27,28] has shown that GFRP fibers improve the toughness of cementitious composites due to the ductility of fiber-reinforced composites. However, the compressive strength was not generally improved due to the relatively weak bonding between cementitious matrices and aggregates [26,28,29]. Besides the research in cementitious composites, it appears that the feasibility of using retired wind turbine blade aggregates has not been investigated for asphalt. Wang et al. [30] reviewed different recycling strategies for decommissioned wind turbine blades. Luo et al. [31] studied the effects of a silane agent on the interfacial performance between recycled wind turbine blades and asphalt binders or SBR. Nie et al. [32] investigated the effects of SBS-modified asphalt on fibers made from recycled wind turbine blades through various experiments. Zhang et al. [33] proposed a method for recycling glass fibers from wind turbine blades using pyrolysis gas combustion and flue gas recirculation. According to the China Aggregates Association, the construction [34], expansion, and maintenance of highways and other roads in China are expected to reach 100,000 km in the next few decades, requiring over 3 billion tons of aggregates. The excavation of aggregates consumes significant amounts of natural mineral resources, and the transportation of these aggregates incurs substantial expenses and carbon emissions [35,36,37,38]. As organic materials, asphalt exhibits good compatibility with aggregates made from glass fiber-reinforced composites due to their intrinsic similarities. How to use retired wind turbine blade aggregates in asphalt pavements needs to be investigated. The amount of retired wind turbine blades to be processed in the near future requires a timely solution, which could be strengthened by diverse applications in pavement. Moreover, the excellent mechanical properties of fiber-reinforced composites need to be retained in recycling because wind turbine blades are designed with superior fatigue resistance under dynamic loads.

Despite prior studies on using GFRP fibers and powders in asphalt binders [29,30,31,32,33], the adhesive properties between asphalt binder and GFRP aggregates remain unclear. Meanwhile, the effects of load and water on the interfacial properties between binders and GFRP have not been elucidated. This becomes a critical challenge in recycling retired wind turbine blades as aggregates when the number of retired wind turbines increases dramatically. The objective of this study is to investigate the interfacial behavior between asphalt and aggregates made from glass fiber-reinforced composites at the microscale, findings from which could provide essential clues to the creation of asphalt mixtures using aggregates from retired wind turbine blades.

This paper first introduces interfacial molecular models of asphalt binders, minerals, and GFRP aggregates. After model predictions are verified by experimental measurements, the chemical adhesion between various asphalt binders and GFRP aggregates is further compared with that between binders and mineral aggregates. Then, the influence of loading rates is examined in terms of how they affected the interfacial behavior between aggregates and asphalt in both dry and hydrated states. Finally, discussions are presented, along with potential research directions.

## 2. Materials and Methods

### 2.1. Materials

#### 2.1.1. Asphalt Binders

**Figure 1:**
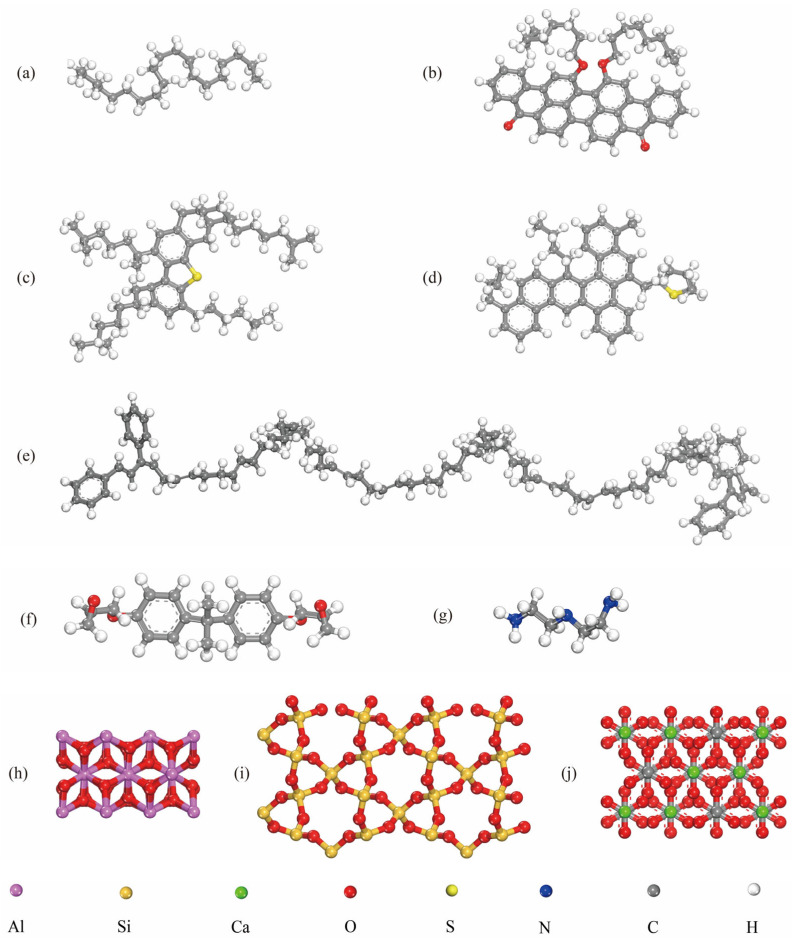
Molecular formulas of (**a**) saturate, (**b**) asphaltene, (**c**) resin, (**d**) aromatic, (**e**) styrene–butadiene–styrene (SBS). (**f**) DGEBA, (**g**) TETA, (**h**) alumina, (**i**) silica, and (**j**) calcite.

In this research, the control binders were AH 70 and PG 64-22. A styrene–butadiene–styrene (SBS) block copolymer was used to modify the AH 70 binder at 2.5 to 6.5 wt% fractions. The complex constituents of asphalt binders were roughly represented by saturates, aromatics, resins, and asphaltenes (SARA) [39]. Different molecular models of asphalt are used in the literature, such as three- and twelve-component molecules. To be consistent with our prior experimental studies [40,41,42,43,44], the four-component SARA model was used in this study. In this study, saturates, aromatics, resins, and asphaltenes are denoted by C_20_H_42_, C_45_H_48_S, C_50_H_80_S, and C_50_H_48_O_4_ [45], respectively. SARA fractions of the AH 70 asphalt binder [46] and the PG 64-22 asphalt binder [46,47] are listed in Table 1. The SARA molecular structures are shown in Figure 1. The chemical formula of styrene–butadiene–styrene (SBS) is C_100_H_128_ [48,49,50,51]. Models with similar sizes were selected for different binders, i.e., 35.0 Å × 35.0 Å × 35.0 Å for AH 70 and 27.4 Å × 27.4 Å × 27.4 Å for PG 64-22.

#### 2.1.2. Aggregates

Aggregates used in this study included mineral aggregates and aggregates made from GFRP composites. Mineral aggregates were represented by silica (SiO_2_), alumina (Al_2_O_3_), and calcite (CaCO_3_) (Figure 1). GFRP composites were made from epoxy matrices and glass fibers (silica). In this study, epoxy was obtained by crosslinking Diglycidyl Ether of Bisphenol A (DGEBA) and triethylenetetramine (TETA) [52]. Chemical formulas of DGEBA and TETA were C_21_H_24_O_4_ and C_6_H_18_N_4_ [53], respectively (Figure 1).

### 2.2. Experiments

**Figure 2:**
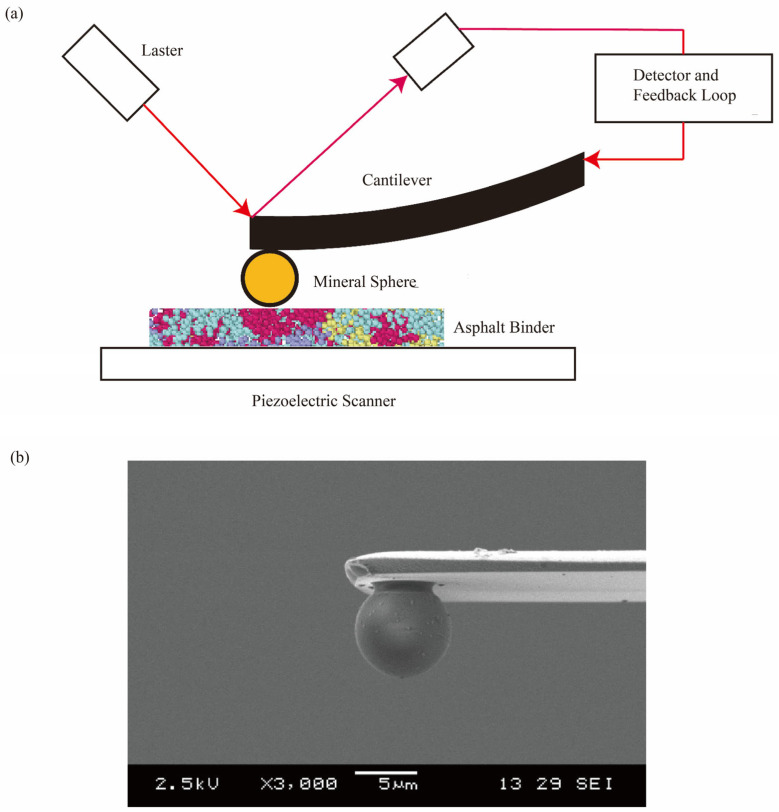
(**a**) A schematic diagram showing the operational principle of PPSFM. (**b**) SEM imaging of a silica microsphere-modified microprobe (**b** reproduced from [56] with permission from Elsevier).

In our prior studies, particle probe scanning force microscopy (PPSFM) was utilized to examine the adhesion between aggregates and asphalt [40,41,42,43,44,54,55,56]. Figure 2a presents a schematic diagram showing the operational principle of PPSFM. The quantification of the adhesion between mineral spheres and asphalt binders was achieved by measuring the deflection of microcantilever beams at the moment of separation. In prior studies [39,40,41,42,43], three types of mineral spheres (each with a diameter of ~5 µm), including silica, alumina, and calcium carbonate, were used to create particle probes by attaching them to the free ends of microcantilevers. Uniform binder substrates were prepared by spreading asphalt binders across wafer surfaces, exhibiting a root mean square (RMS) surface roughness of ~30 nm. Further details of experiments are provided in [40,41,42,43,44]. Figure 2b shows an SEM image of microcantilever beam with an attached silica microsphere. By using PPSFM, we successfully distinguished the adhesion between various virgin and modified asphalt to mineral spheres [40,41,42,43,44]. In this study, adhesion measurements between PG 64-22 binder (Library, MRL, Austin, TX, USA) and silica (Microspheres-Nanospheres Inc., Cold Spring, NY, USA) were used to verify the predictions from molecular simulations.

### 2.3. Molecular Modeling

Molecular dynamics were performed using LAMMPS (Large-scale Atomic/Molecular Massively Parallel Simulator) to elucidate the molecular adhesive mechanisms at dry and hydrated interfaces between asphalt binders and aggregates. The force fields and simulation details are explained in subsequent sections.

#### 2.3.1. Forcefields

The Consistent Valence Forcefield (CVFF) was employed to model the interfacial interactions between aggregates and asphalt binders [57,58,59,60,61]. In the numerical simulation, $E_{Total}$ representing the total potential energy was determined by

(1) $$E_{Total}={E_{Bond}+E}_{Angle}+E_{Dihedral}{+E_{Improper}+E}_{Pair}{+E}_{Coulomb}$$

where $E_{Bond}$, $E_{Angle}$, $E_{Dihedral}$, and $E_{Improper}$ are contributions from stretching and compression for bonds, valence angle bending, internal rotation, or torsion for dihedral anglesand (out-of-plane) improper angles. $E_{Pair}$ and $E_{Coulomb}$ are contributions from van der Waals and Coulomb interactions, respectively.

#### 2.3.2. Interfacial Components in Molecular Models

All molecular structures were constructed using Materials Studio^TM^ software (version 2024). Perl scripts were used to connect DGEBA and TETA, resulting in an epoxy with the crosslinking degree of 73.3%. Silica, alumina, and calcium carbonate were constructed based on crystal structures of α-quartz, corundum, and calcite [62,63,64]. SARA components and the SBS modifiers were incorporated into asphalt models using amorphous cell modules. To create AH 70 and PG 64-22 binders, SARA fractions were mixed at the weight fractions listed in Table 1. Meanwhile, modified asphalt binders were prepared by numerically mixing AH 70 binder and SBS at different weight fractions from 2.5 to 6.5 wt%.

**Figure 3:**
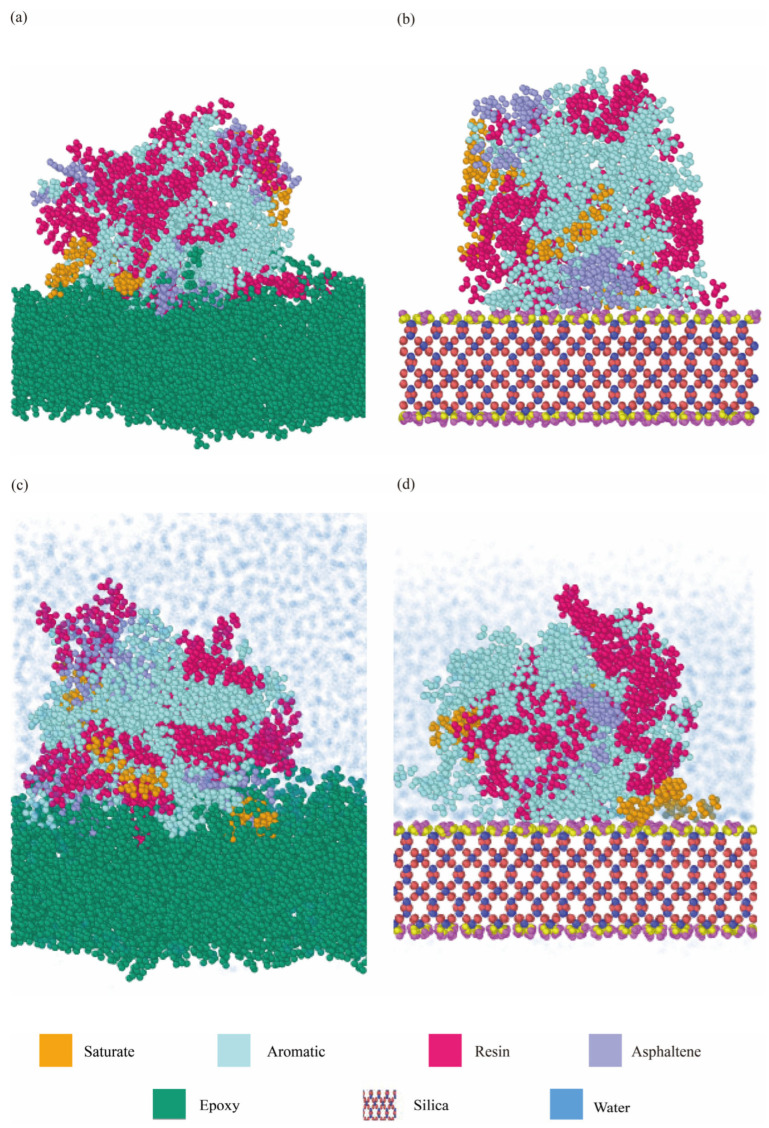
Representative molecular models of interfaces between (**a**) the AH 70 binder and epoxy in the dry condition and (**b**) the AH 70 binder and silica in the dry condition. (**c**) The AH 70 binder and epoxy in the hydrated condition. (**d**) The AH 70 binder and silica in the hydrated condition. Blue dots indicate water molecules in (**c**,**d**).

Representative molecular models between various asphalt binders and aggregates are shown in Figure 3, including dry and hydrated interfaces between AH 70 asphalt binder and silica, and dry and hydrated interfaces between AH 70 binder and epoxy [65]. Four types of aggregate substrates, i.e., epoxy, alumina, silica, and calcite, were created at sizes of 85~120 Å × 85~120 Å × 17~30 Å (Table 2).

To study how water affects the interfacial behavior between aggregates and binders, the aggregate–binder adhesion was assessed by immersing the entire system in water [66,67,68]. Approximately 7000 water molecules were added to enclose the binders and the interfaces. In this study, models in the dry condition included interfaces between the AH 70 binder and epoxy and interfaces between the AH 70 binder and silica (Figure 3a,b). Models in the hydrated condition included interfaces between the AH 70 binder and epoxy and interfaces between the AH 70 binder and silica. In Figure 3c,d, water molecules are represented by blue dots. In this study, the numbers of atoms in molecular models ranged from ~15k and ~28k.

#### 2.3.3. Modeling Setup

In this study, molecular simulations were conducted by employing LAMMPS software (version stable_29Aug2024) [69]. Interatomic interactions were modeled using a 10 Å cutoff Lennard–Jones potential. The electrostatic forces were computed using the Ewald sum technique [70]. Using the Nose–Hoover thermostat and barostat, the model systems were equilibrated at 300 K and 1.0 atm and integrated at 1 fs time steps.

Subsequently, the system energy was minimized using the conjugate gradient (CG) approach. Interfacial systems were stabilized at 300 K in the canonical ensemble (NVT) for 500 picoseconds, and then in the isothermal–isobaric ensemble (NPT) for 100 picoseconds. When the bottoms of interfacial models were constrained, these models were further equilibrated for 300 picoseconds in the NVT ensemble.

**Figure 4:**
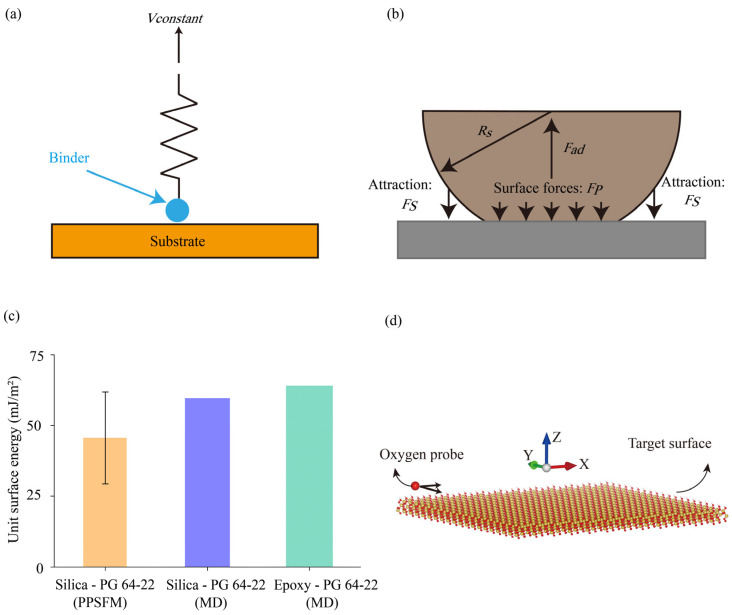
Schematic diagrams illustrating (**a**) the SMD method. (**b**) The DMT model. (**c**) Comparison of unit surface energy measurements between PG 64-22 binder and silica using PPSFM and predictions from molecular modeling. Experimental data were obtained from our prior studies in [36]. Predicted unit surface energy (blue and green bars) were calculated via molecular simulations. (**d**) A schematic diagram of the PES calculation in this study.

In the literature, steered molecular dynamics (SMD) was used to determine the forces exerted on specific atomic groups in multiple processes [71,72,73]. By applying a gradual pulling force to a spring tethered to an atom within the molecular group, asphalt binders were progressively separated from the interfaces using the SMD technique. The schematic diagram of SMD is shown in Figure 4a.

This study planned to elucidate how different loading rates affected aggregate–binder adhesion under normal and extraordinary conditions that are difficult to examine in experiments. Thus, asphalt binders were pulled from mineral substrates at speeds ranging from traffic speeds (2–100 m/s) to extreme impact or blast scenarios (100–800 m/s) [62,74,75,76,77,78,79,80]. In simulations, a spring constant of 1.34 N/m was utilized, which was aligned with the spring constants (1–5 N/m) from prior adhesion measurements [42,43,81].

## 3. Analytical Models

### 3.1. Unit Surface Energy

The unit surface energy serves as an effective measure for asphalt binder adhesion [82,83], which was calculated using the Derjaguin–Muller–Toporov (DMT) theory in the literature [40,41,42,43,71]. In the DMT model, the deformation was simulated using Hertzian contact and additional interactions from van der Waals in neighbor areas. A schematic diagram of the DMT model is shown in Figure 4b. The unit surface energy *φ* is given by

(2) $$\varphi =\frac{F_{ad}}{4\pi R_{s}}$$

where $F_{ad}$ is the adhesive force between aggregates and binders, and $R_{s}$ is the radius of the contact sphere.

### 3.2. Bell’s Theory

Bell’s theory was proposed to examine pull-off forces between different solids at various loading rates [84]. The model was used to describe the adhesion between different solid interfaces [74,85,86].

(3) $$\nu =v_{0}\cdot \exp\left(\frac{f\cdot x_{b}}{k_{b}\cdot T}\right)$$

where f is the pulling force. $v_{0}$ is the bond-breaking speed. $x_{b}$ quantifies the separation distance between the bound state and the maximum energy value. $E_{b}$ is the energy barrier. $k_{b}$ is the Boltzmann constant.

(4) $$v_{0}=\omega_{0}\cdot x_{b}\cdot \exp\left(-\frac{E_{b}}{k_{b}\cdot T}\right)$$

The combination of Equations (3) and (4) and yields Equation (5).

(5) $$f=\frac{k_{b}\cdot T}{x_{b}}\cdot \ln\left(v\right)-\frac{k_{B}\cdot T\cdot \ln\left(v_{0}\right)}{x_{b}}=A\cdot \ln\left(v\right)+B$$

where *A* and *B* denote fitting constants.

### 3.3. Adhesive Strength

Adhesive strength between binders and aggregates was obtained by dividing the adhesive force over the initial cross-sectional area of the interface, given by

(6) $$\sigma =\frac{F_{ad}}{A_{S}}$$

where σ is the adhesive strength, and $A_{S}$ is the initial cross-sectional area of the aggregate–binder interfaces. $F_{ad}$ is the adhesive force between aggregates and binders.

### 3.4. Radial Distribution Function (RDF)

The radial distribution function *g*(*r*) quantifies the probability of finding a particle at a distance *r* from another particle in the microstructure, given by

(7) $$g(r)=\underset{\mathrm{dr}\to 0}{\lim}\frac{dN}{\rho 4\pi r^{2}dr}$$

where N is the number of particles in the system, dN is the number of specified particles at a distance r to r+dr, and *ρ* is the average density of the system.

### 3.5. Interfacial Morphology and Potential Energy Between Asphalt and GFRP

The root mean square (RMS) was utilized to describe the surface roughness of aggregates and binders. The RMS of surface morphology surfaces ($R_{h}$) was calculated by

(8) $$R_{h}=\sqrt{\frac{\sum_{n=1}^{N}{\left(h_{i}-h_{a}\right)}^{2}}{N}}$$

where $h_{i}$ is the height in position i on the target surface, $h_{a}$ is the average height of the target surface, and N is the number of points selected.

Meanwhile, the RMS of potential energy ($R_{e}$) was calculated by

(9) $$R_{e}=\sqrt{\frac{\sum_{n=1}^{N}{\left(E_{i}-E_{a}\right)}^{2}}{N}}$$

where $E_{i}$ is the potential energy in position i, $E_{a}$ is the average potential energy, and N is the number of points selected.

## 4. Results and Discussions

### 4.1. Verification of Adhesion Predictions from Molecular Modeling

Computational predictions from molecular modeling were benchmarked against experimental findings [61,63,64,65,70,71]. The unit surface energy between PG 64-22 binder and silica is compared in Figure 4c. It was shown that experimental measurements obtained from PPSFM were lower than predicted values from molecular simulation. This was owing to the size differences of microspheres between the experiment and models. The diameter of silica microsphere was ~6 μm, whereas diameters of mineral substrates in molecular models were ~100 nm. Larger contact areas with more interfacial defects could result in lower adhesion in experiments relative to those in molecular simulations. It was also shown that the predicted interfacial adhesion between PG 64-22 binder and epoxy was higher than that between PG 64-22 binder and silica. Generally, the models exhibited good agreement to adhesion measurements.

### 4.2. Interfacial Behavior from Molecular Modeling

#### 4.2.1. Morphologies of Interfaces Between Aggregates and Binders

Interfacial morphologies between aggregates and binders are critical to their adhesion [87,88]. It is challenging to examine intact morphologies of adhered asphalt binders and aggregates through non-destructive experiments. In this study, the authors examined interfacial morphologies using potential energy surfaces (PES). To calculate the potential energy surfaces, a single uncharged oxygen atom was moved along the target surface in both X and Y directions to scan over the target surfaces [89,90], i.e., the asphalt binder or aggregate surfaces when adhered. A schematic diagram showing the PES calculation is shown in Figure 4d. As the scan proceeded, the minimum potential energy (calculated from the potential energy term resulting from van der Waals force in Equation (1)) and associated altitudes of oxygen probe were recorded to construct the potential energy contours, which represented three-dimensional configurations of target surfaces, i.e., asphalt binder and aggregate surfaces when adhered. The coloration of the potential energy surface illustrated the energy levels of local regions among the entire adhered surfaces.

**Figure 5:**
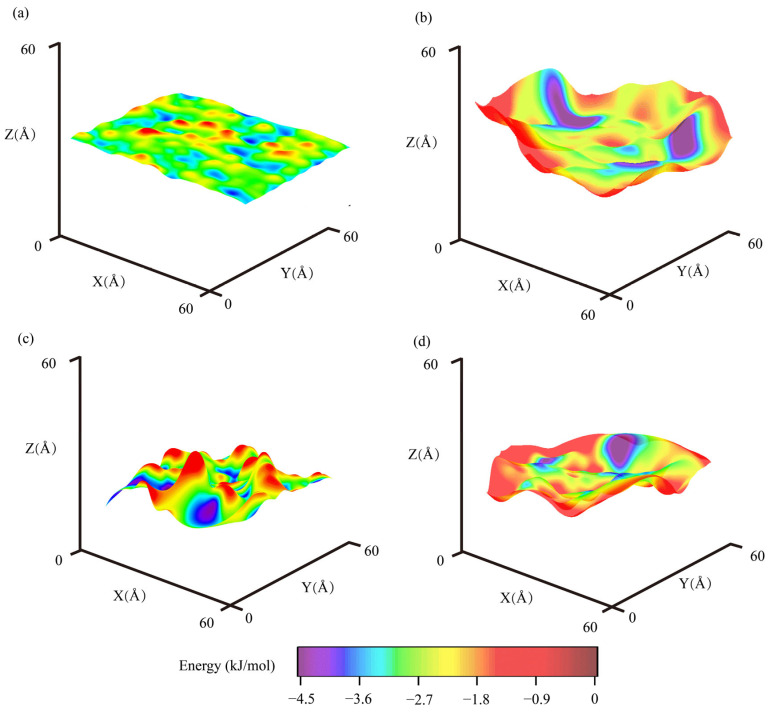
Potential energy surfaces between adhered asphalt binders and substrate. Morphologies of dry interfaces between (**a**) silica and (**b**) AH 70 binder. Morphologies of hydrated interfaces between (**c**) epoxy and (**d**) AH 70 binder.

In this study, PES were plot for different asphalt aggregate−binder interfaces, including interfaces between AH 70 and silica or epoxy in the dry state (Table 3). When AH 70 and silica were adhered (Figure 5a,b), the surface morphologies of asphalt binders were rougher than their adhered mineral pairs, such as silica (*R_h_* = 0.44 Å) and the mate asphalt (*R_h_* = 1.44 Å). This was probably because amorphous structures of polymer chains in asphalt binders were more flexible than the crystal structures of silica. In contrast, the epoxy was an amorphous polymer whose surface morphology (*R_h_* = 3.30 Å) was close to those of asphalt binders (*R_h_* = 1.98 Å), as shown in Figure 5c,d. These rough surfaces could lead to improved interactions between asphalt binders and epoxy matrices, as demonstrated in prior sections, where epoxy was shown to exhibit greater adhesion to binders than silica. Meanwhile, for the interface between AH 70 binder and silica, the mean potential energy of silica (−2.62 kJ/mol) was similar to that of the binder (−2.23 kJ/mol). For the interface between epoxy and AH 70 binder, the mean potential energy of epoxy (−2.49 kJ/mol) was similar to that of the binder (−1.99 kJ/mol). The *R_e_* of different interfaces were relatively close (~1 kJ/mol), as shown in Table 3.

It should be emphasized that PES in this study showed morphologies of aggregate–binder interfaces at the nanoscale, whereas interfaces at larger scales included more defects and complex structures. Despite the differences, this visualization could further our understanding of how adhered binders and aggregates stay with each other.

#### 4.2.2. Radial Distribution Functions (RDF) of Binder Components

**Figure 6:**
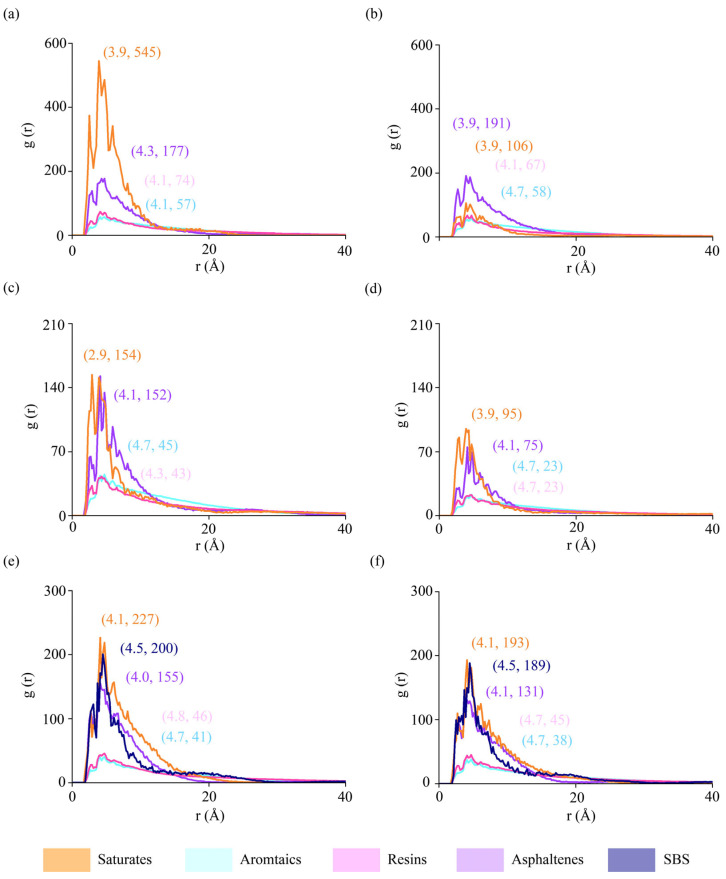
Radial distribution functions of different binder components. Interfaces between silica and AH 70 binder (**a**) in the dry condition and (**b**) in the hydrated condition. Interfaces between epoxy and AH 70 binder (**c**) in the dry condition and (**d**) in the hydrated condition. Interfaces between epoxy and AH 70 binder with 4.5% SBS (**e**) in the dry condition and (**f**) in the hydrated condition.

Radial distributions were plotted to examine the local packing densities of asphalt components around asphalt–aggregate interfaces (Figure 6). RDF values were calculated when molecular models were completely settled after the relaxation of ~900 picoseconds. The radius of 40 Å was selected to cover the entire molecular model, and the thickness of the slice was 0.2 Å. Six interfacial pairs were presented, including dry and hydrated interfaces between AH 70 binder and silica, between AH 70 binder and epoxy, and between AH 70 with 4.5% SBS and epoxy.

In Figure 6a to Figure 6b, the peaks of saturates decrease significantly, whereas peaks of other components remain at similar levels. This shows that water molecules effectively affected the distributions of saturates at aggregate–binder interfaces. At interfaces between AH 70 and epoxy (Figure 6c,d), RDF curves showed that almost all peaks of SARA components changed in dry and hydrated cases, meaning that the polymeric interfaces between epoxy and asphalt binders changed coordinately to water molecules than silica–binder interfaces. When SBS was added into AH 70 (Figure 6e,f), peaks in SARA fractions and SBS were similar in the dry and hydrated cases, which was because the modifier helped stabilize microstructures of asphalt binders. In general, the inclusion of water and modifiers affected the microstructures of components in asphalt binders compared to those in unmodified, dry conditions.

**Figure 7:**
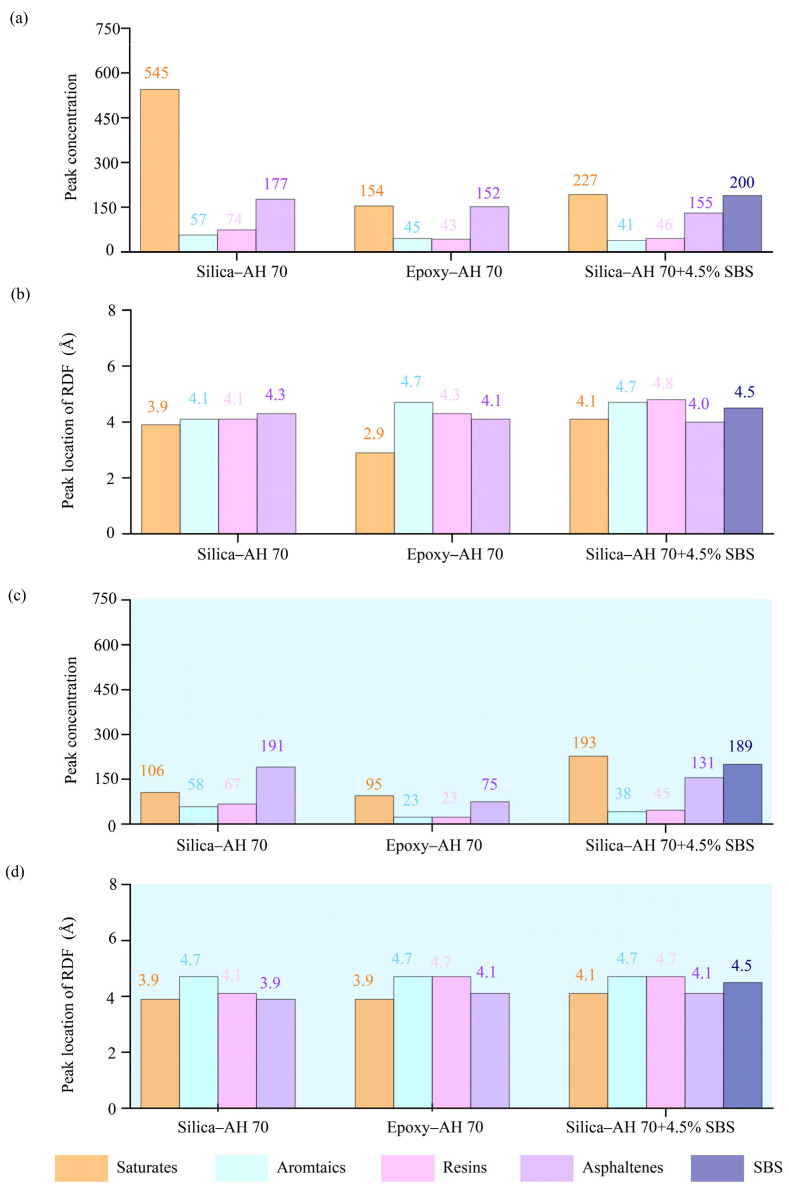
Radial distribution function peaks and locations of SARA components at silica–AH 70, epoxy–AH 70, and epoxy–AH 70 with 4.5% SBS in the (**a**,**b**) dry and (**c**,**d**) hydrated conditions.

For radial distribution functions, peak values and the corresponding positions away from interfaces are presented for SARA components in Figure 7. Results revealed that saturates and asphaltenes exhibited significantly higher peak RDF values than aromatics and resins. The introduction of water lowered peak RDF values for SARA components and SBS. Locations of peak RDF values were similar for the epoxy-based system in the dry and hydrated cases (Figure 7b,d).

#### 4.2.3. Spatial Distributions of Binder Components

**Figure 8:**
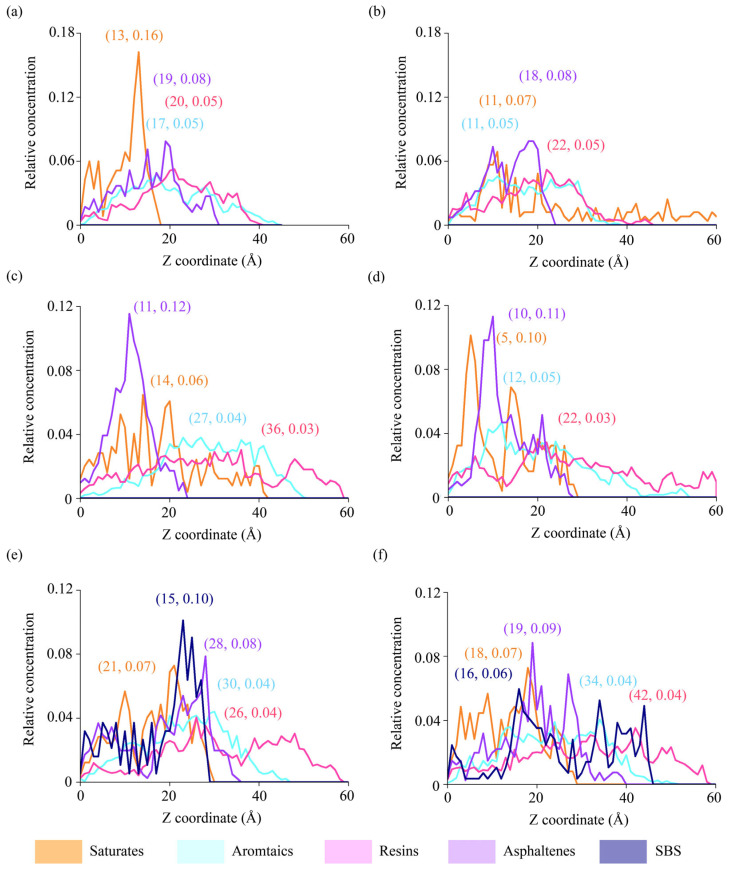
Relative concentrations of asphalt components away from interfaces. Interfaces between silica and AH 70 (**a**) in the dry and (**b**) in the hydrated conditions. Interfaces between epoxy and AH 70 (**c**) in the dry and (**d**) hydrated conditions. Interfaces between epoxy and AH 70 with 4.5% SBS (**e**) in the dry and (**f**) hydrated conditions.

Spatial distributions of asphalt components are plotted away from asphalt–aggregate interfaces (Figure 8). Six interfaces in Section 4.2.2 were selected again for the analysis of relative concentrations. Regarding the interface between AH 70 binder and silica, the concentration peaks of saturates in the dry condition occurred close to the interface (Figure 8a), whereas the concentration peaks of saturates diminished in the hydrated condition (Figure 8b). This showed that water molecules affected the spatial distributions of saturates, which is also shown in Section 4.2.2. In Figure 8a,b, the spatial distributions of aromatics, resin, and asphaltene were relatively stable. The elongated chain structures of saturates made them more susceptible to water molecules.

However, the spatial distributions of binders at interfaces between epoxy and asphalt were different. In both dry and hydrated cases, asphaltene exhibited large concentration peaks close to the aggregate–asphalt interfaces (Figure 8c,d). Meanwhile, the introduction of water enabled the concentration peaks of saturates in the hydrated conditions to be higher than those in the dry conditions and closer to the interfaces, which were different from the trends observed in silica–asphalt interfaces (Figure 8a,b). When SBS was added to modify the virgin asphalt binders (Figure 8e,f), the spatial distributions of four SARA components were very similar in both dry and wet cases, showing that the SBS modifier helped stabilize the binder components. However, the concentration peaks of SBS were higher in dry than in hydrated cases, meaning that the spatial distributions of SBS could be affected by water.

**Figure 9:**
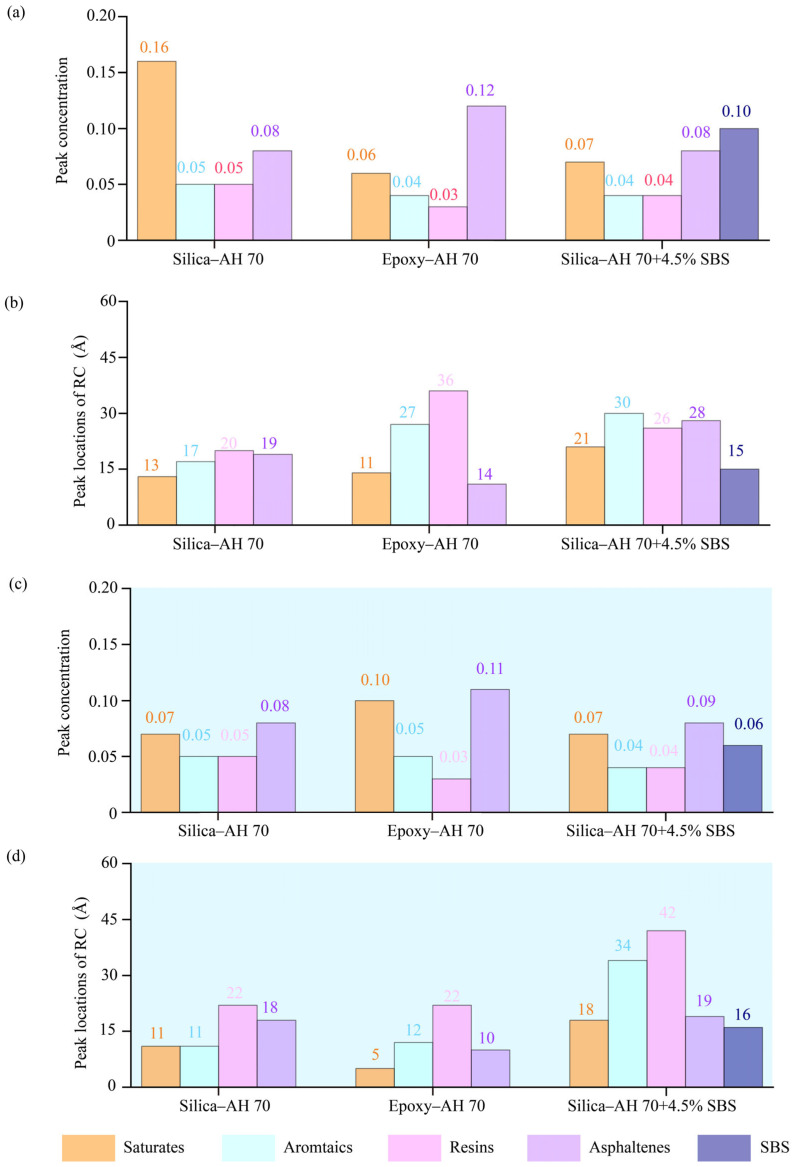
Relative concentration peaks and locations of SARA components at silica–AH 70, epoxy–AH 70, and epoxy–AH 70 with 4.5% SBS in the (**a**,**b**) dry and (**c**,**d**) hydrated conditions.

Regarding relative concentrations, peak values and the corresponding positions away from interfaces are presented for SARA components in Figure 9. Results revealed that saturates and asphaltenes exhibited significantly higher peak RC values than aromatics and resins in both the silica-based and epoxy-based systems. SBS reduced the disparity of RC peaks, improving the uniform distributions of SARA components. In the silica–SBS systems, the addition of water enlarged locations of peak RC values for aromatics and resins, but lowered locations of peak RC values of saturates and asphaltenes (Figure 9b,d).

#### 4.2.4. Load–Displacement Curves Between Asphalt Binders and Epoxy

**Figure 10:**
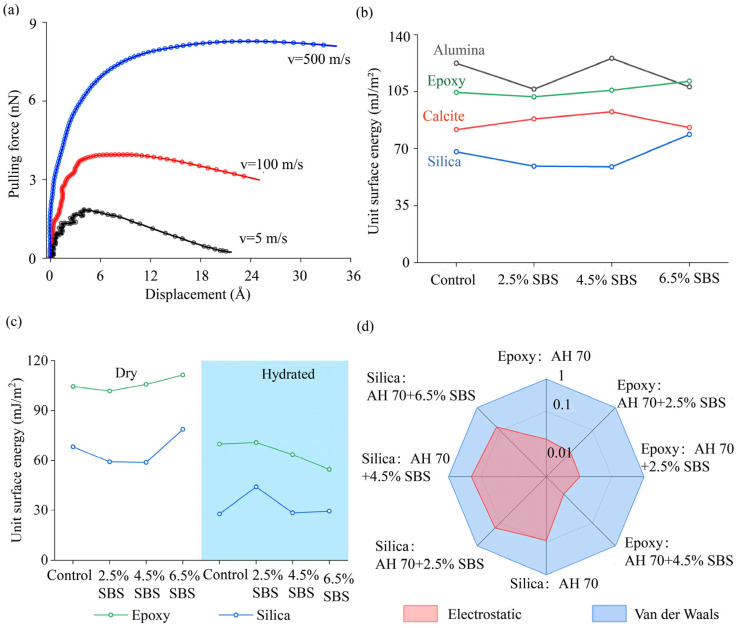
(**a**) Pulling forces versus displacements at different loading rates between the AH 70 asphalt binder and epoxy substrates. Three curves were generated at 5 m/s, 100 m/s, and 500 m/s, respectively. (**b**) Predicted adhesion between epoxy or mineral aggregates and SBS-modified AH 70 binders in dry conditions. (**c**) Predicted adhesion between epoxy or silica and SBS-modified AH 70 binders in the dry and hydrated conditions. (**d**) Contributions from electrostatic and van der Waals forces to adhesion between binders and aggregates.

Using the SMD method, asphalt binders were pulled by linking one end of the stretching spring to the binder molecules at loading rates between 2 and 800 m/s. Representative load–displacement curves are shown (Figure 10a) for the pulling of the AH 70 binder away from the epoxy substrate at 5, 100, and 500 m/s. Larger peak adhesive forces and maximum pulling distances were obtained between asphalt binders and epoxy with increased loading rates.

#### 4.2.5. Adhesion Between GFRP Aggregates and Modified Binders

To examine interactions between GFRP aggregates and modified binders, adhesion between minerals and different SBS-modified AH 70 asphalt binders was investigated using molecular simulations. The epoxy was selected to represent the matrix in GFRP composites. Alumina, silica, and calcite were chosen to represent natural aggregate minerals. The AH 70 binder was modified by three SBS concentrations, i.e., 2.5 wt%, 4.5 wt%, and 6.5 wt%. It was found that adhesion between epoxy and the modified binders was greater than that between silica or calcite and corresponding binders, but lower than that between alumina and associated binders in the dry conditions (Figure 10b). These results were consistent with experimental findings in prior studies, in which adhesion at alumina–binder interfaces were higher than those at silica–/calcite–binder interfaces [43,53,54,55]. The adhesion between these minerals and binders varied to a small extent at the selected SBS spectrum (0 to 6.5 wt%). Predicted adhesion between epoxy or silica and SBS-modified AH 70 binders in the dry and hydrated conditions is shown in Figure 10c. It was shown that the epoxy–asphalt adhesion was higher than silica–asphalt in both dry and hydrated cases.

These results proved that the affinity between epoxy and asphalt binders at the nanoscale was comparable to that between mineral aggregates and their counterparts. However, these findings were deviated from prior experimental results from uniaxial penetration tests [91], in which lower adhesion was observed between macroscale GFRP aggregates and asphalt binders than between mineral aggregates and binders. This was because smooth surfaces of GFRP aggregates generated fewer interlocking effects to asphalt binders than rough surfaces of mineral aggregates, although epoxy was chemically adhesive to organic components of asphalt binders. Further work will be performed to reveal how surface textures of GFRP aggregates affect their adhesion to various asphalt binders.

Moreover, aggregate–binder adhesion was further analyzed by quantifying contributions from electrostatic and van der Waals forces. It was found that van der Waals forces contributed dominantly (>80%) to aggregate–binder adhesion (Figure 10d). The contribution from van der Waals to epoxy–binder adhesion was greater than that to mineral–binder adhesion. These results suggested that aggregates with high surface roughness helped to increase the adhesion between asphalt binders and aggregates, which were consistent with our strategy for material selection in asphalt mixtures.

#### 4.2.6. Loading Rates Versus Interfacial Adhesion

**Figure 11:**
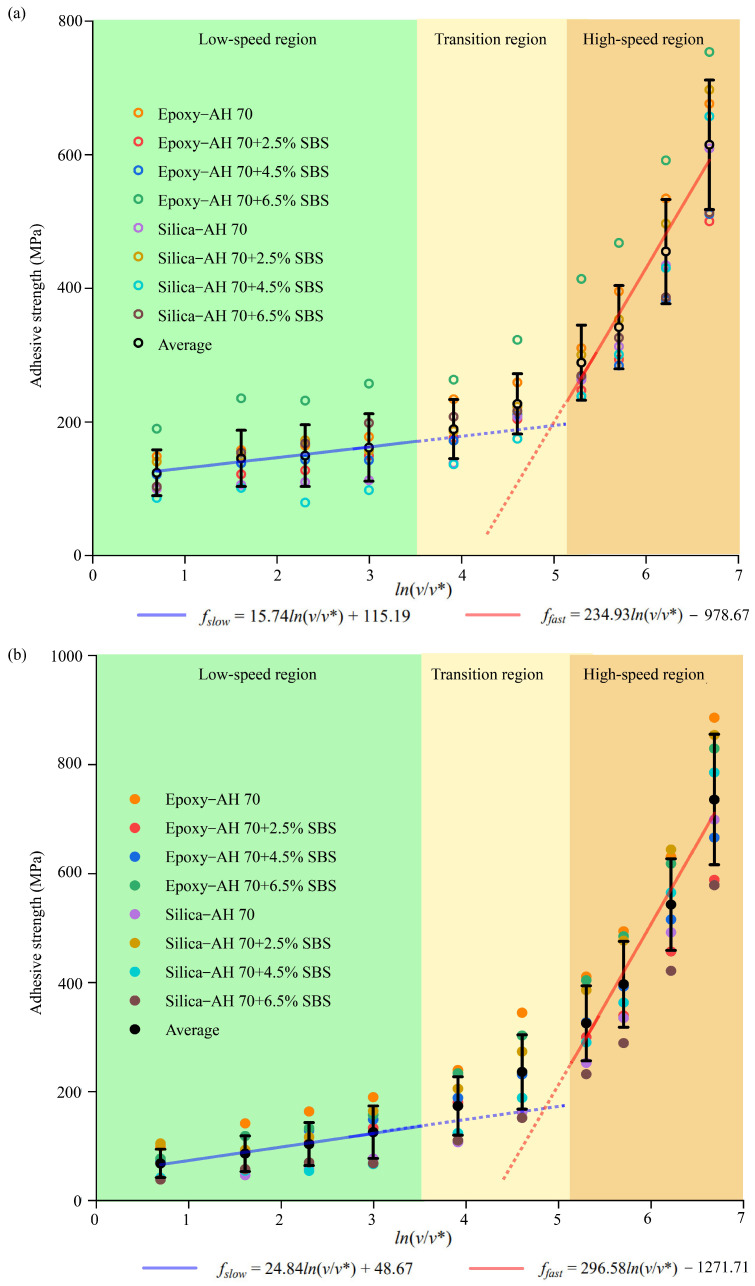
Adhesive strength at different loading rates. Interfaces between various SBS-modified AH 70 binders and epoxy or silica (**a**) in dry conditions and (**b**) in hydrated conditions. (A reference velocity of 1 m/s ($v^{*}$ was adopted for rate normalization.)

Adhesive strengths for different loading rates were compared between SBS-modified binders and silica or epoxy (Figure 11). Distinctive regions existed at different loading rates. In the low-speed region (<50 m/s), a relatively flat line was fitted to the strength rate data. However, the fitting line was much elevated to accommodate the rapid growth of adhesive strength at the high-speed region (>200 m/s). A transition region existed in between without obvious trends, which was related to the alteration of microstructural responses at aggregate–binder interfaces with elevated loading rates. In the same vein, adhesive strengths between aggregates and various SBS-modified AH 70 binders in hydrated conditions were plotted against loading rates in Figure 11b. Three distinctive regions were also observed. This was consistent with trends described by Bell’s theory [84].

At low loading rates, sufficient time was available for interfaces between aggregates and binder to respond and to rupture, resulting in lower adhesion. However, at high rates of loading, simultaneous breakage of multiple bonds at aggregate–asphalt interfaces created large adhesive forces in a short amount of time. Once the interfacial energy barrier was surmounted, aggregate–binder interfaces altered the responses to the external loading.

**Figure 12:**
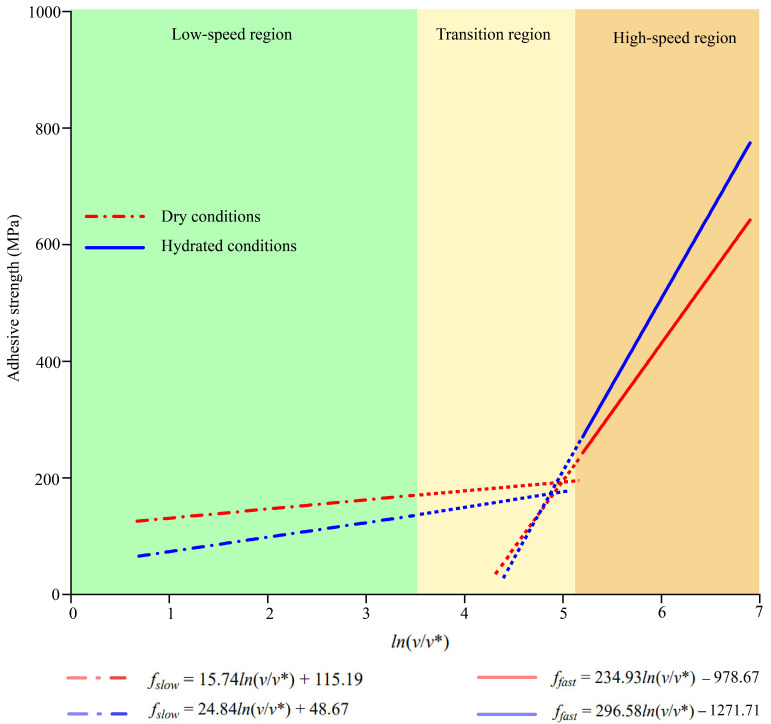
Summary of the relationship between loading rates and adhesive strength between dry and hydrated interfaces.

In the hydrated conditions, distinctive regions also existed for interfaces between silica or epoxy and AH 70 binders (Figure 11b). However, the fitting equations for the dry and hydrated cases were distinct from each other in these regions (Figure 12). At low loading rates, adhesive strength in the dry conditions was higher than that in the hydrated conditions. This was because water molecules entered the interfaces slowly, leading to the progressive delamination between binders from aggregates. At high loading rates, adhesive strength in the dry conditions was lower than that in the hydrated conditions. The reason was that a group of water molecules was pulled along with asphalt binders during delamination, resulting in extra pulling forces within the limited debonding period. This is further shown in later sections.

#### 4.2.7. Debonding Between Aggregates and Binders

**Figure 13:**
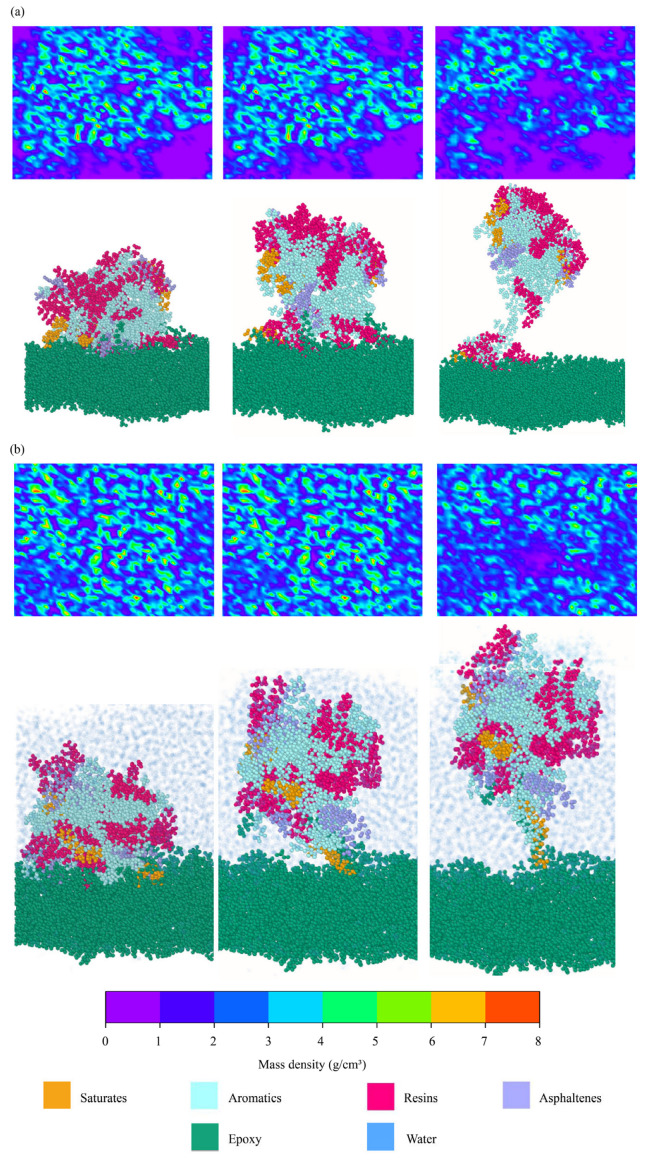
Evolutions of density maps between asphalt binders and epoxy substrates during interfacial delamination at 20 m/s. Interfaces between AH 70 binder and epoxy (**a**) in the dry and (**b**) hydrated conditions.

Density maps were created to further examine the interfacial debonding between binders and aggregates. Four interfacial models were selected, i.e., the dry and hydrated interfaces between AH 70 binder and epoxy and the dry and hydrated interfaces between AH 70 binder and silica. Atomic distributions within the range of 5 Å above and below the interfaces were plotted with different colors, representing the densities of molecules in this region. In Figure 13a,b, the first row shows the top view of the evolution of density maps when the asphalt binder was pulled away from epoxy substrates at 20 m/s. The second row shows the corresponding side view of interfacial delamination listed above. In the dry conditions, when the asphalt binder was pulled away from the epoxy substrate, the epoxy substrate was exposed gradually (Figure 13a). However, blue areas in density maps during the delamination covered more areas of epoxy substrates in the hydrated condition than those in the dry condition (Figure 13b). Water molecules filled the spaces around the binders, resulting in higher atomic densities in the interfacial regions.

**Figure 14:**
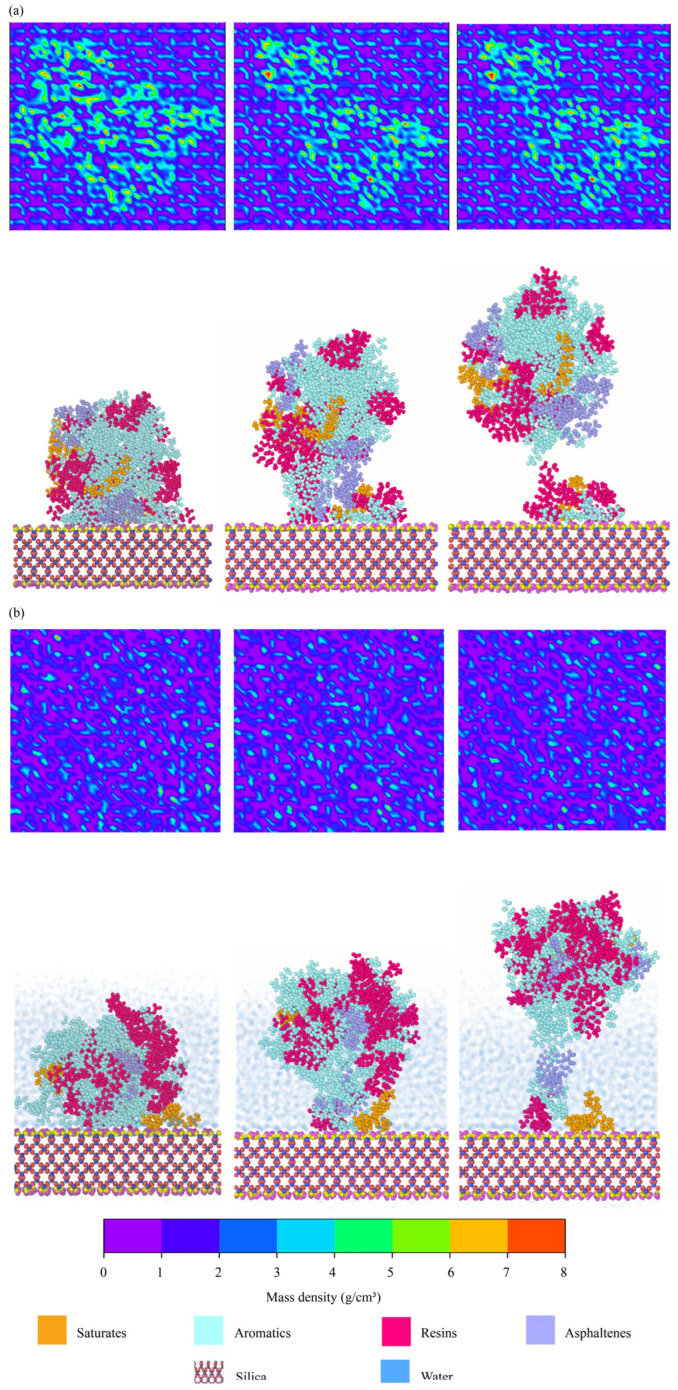
Evolutions of density maps between asphalt binders and silica substrates during interfacial delamination at 20 m/s. Interfaces between AH 70 binder and silica (**a**) in the dry and (**b**) hydrated conditions.

In the same vein, top and side views of delamination between AH 70 binders and silica interfaces are presented in Figure 14. In the dry conditions, similar interfacial areas were observed between epoxy–binder and silica–binder pairs in Figure 13a and Figure 14a. As asphalt binders delaminated from the substrates, limited adhesive regions remained at silica–binder interfaces. In the hydrated conditions, density maps at the silica–binder interfaces were similar from the beginning to the end of delamination (Figure 14b). This was because water molecules propagated into the silica–binder interfaces generated similar density maps during delamination.

#### 4.2.8. Evolutions of Water Molecules During Interfacial Delamination

This section studies the evolutions of water molecules at interfaces when asphalt binders were pulled away from aggregates in the hydrated conditions. To elucidate how water molecules propagated into aggregate–binder interfaces, the number of water molecules and hydrogen bonds were quantified during interfacial delamination. Hydrogen bonds were calculated by checking the bond length (3.5 Å) and bond angle (40°) between hydrogen and the oxygen atoms, which were used in the literature [92,93]. Meanwhile, the number of water molecules at aggregate–binder interfaces was tracked accordingly.

**Figure 15:**
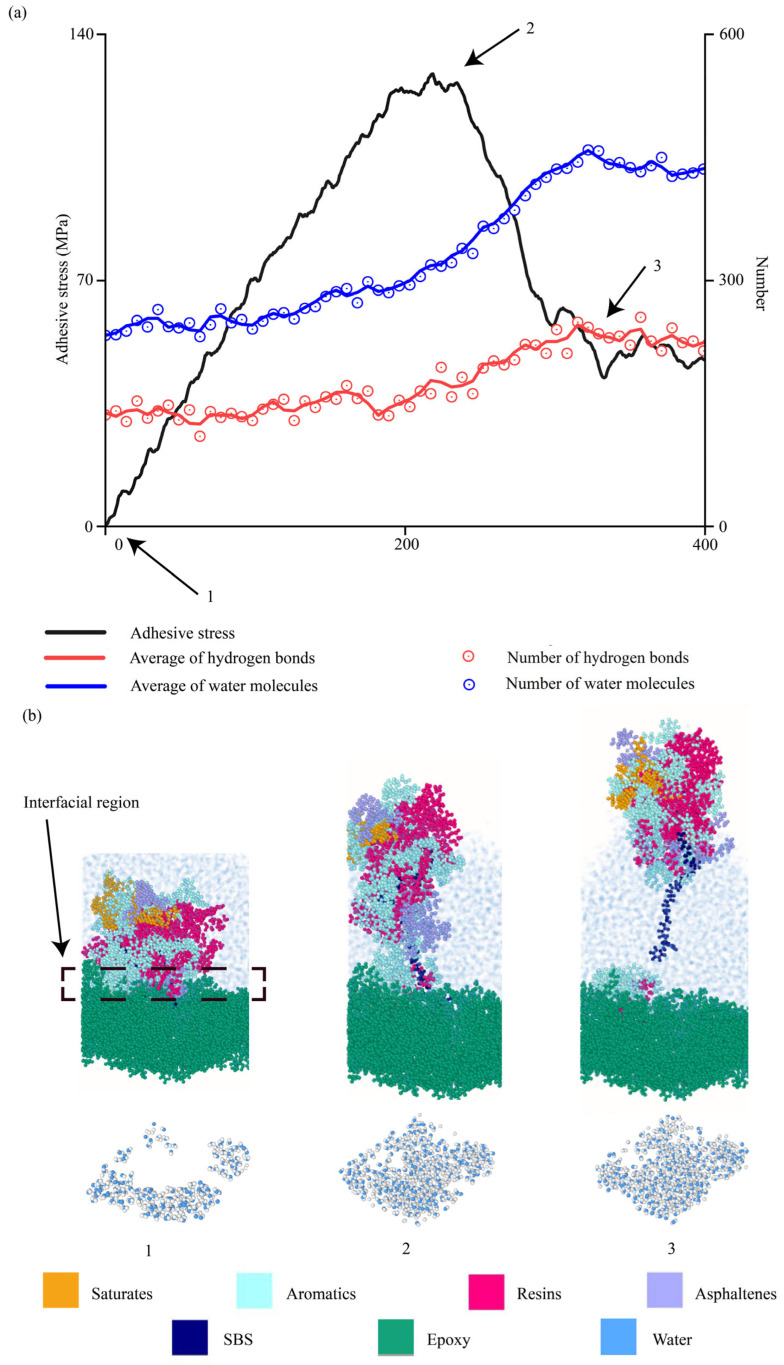
Interfacial evolutions when the AH 70 binder with 4.5% SBS was pulled away from the epoxy substrate at 10 m/s. (**a**) Evolutions of adhesion strength, water molecules, and hydrogen bonds over time. (**b**) Snapshots.

Two delaminations in the hydrated conditions were illustrated, i.e., the AH 70 binder with 4.5% SBS pulled away from the epoxy at 10 m/s and 100 m/s. At 10 m/s (Figure 15), the number of water molecules (blue) and hydrogen bonds (red) increased gradually in the beginning. The numbers increased faster around the peak force when most of the binder left the aggregate surface (Figure 15a). When the binder was separated from the aggregate surface, both the number of water molecules and hydrogen bonds remained stable because water molecules filled the space between the asphalt binder and substrates. Three snapshots, i.e., 1, 2, and 3, were selected to examine the delamination details (Figure 15b). As the modified AH binder was pulled away, water molecules (light blue dots) filled in between. This was further shown by the evolution of water molecules in interfacial regions (shown by clusters of water molecules at the bottom). In the beginning (snapshot 1), water molecules are located around the asphalt binders. Then, more molecules enter the interfacial region (snapshots 2 and 3), which eventually become stable. The white spots in snapshots 2 and 3 show the binder residues on the aggregate surface after delamination.

**Figure 16:**
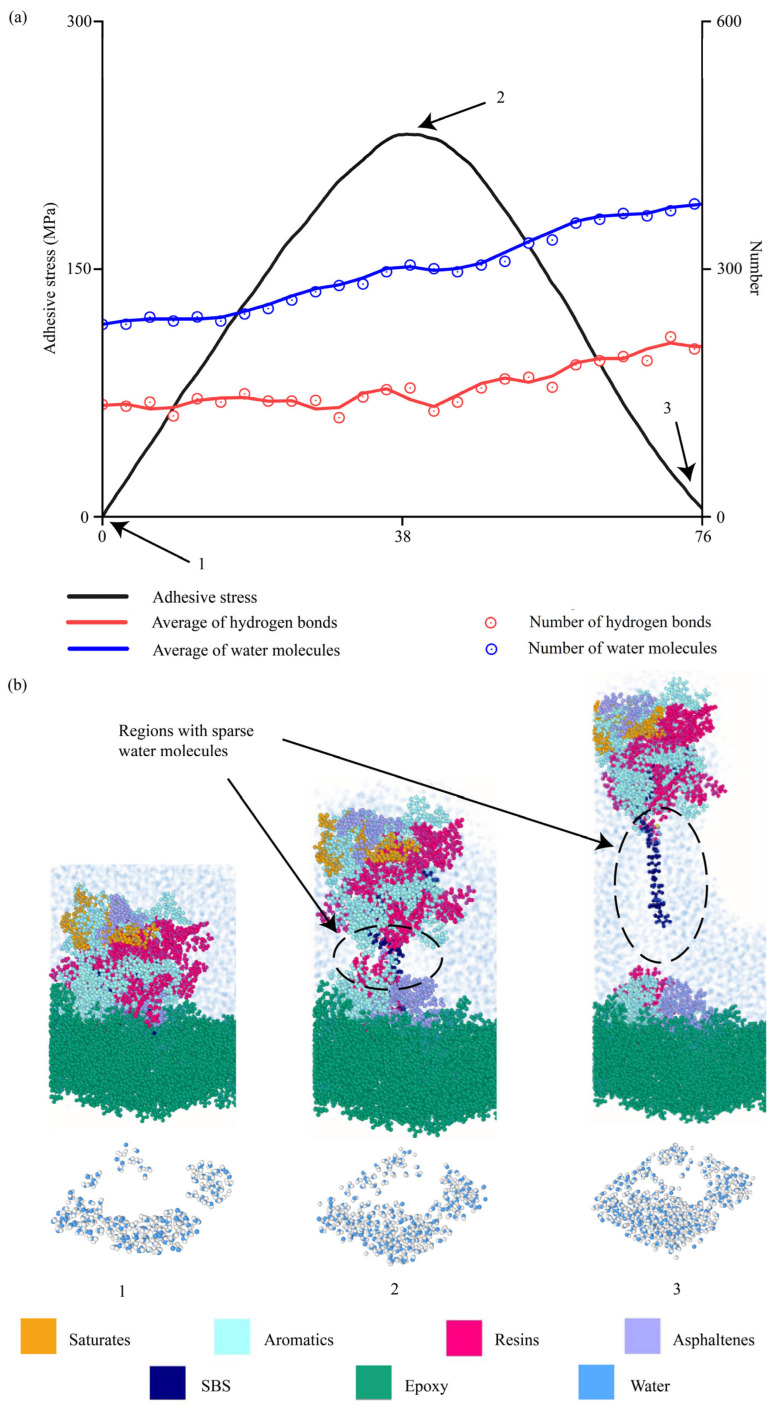
Interfacial evolutions when the AH 70 binder with 4.5% SBS was pulled away from the epoxy substrate at 100 m/s. (**a**) Evolutions of adhesion strength, water molecules, and hydrogen bonds over time. (**b**) Snapshots.

At 100 m/s (Figure 16), water molecules also propagated into the aggregate–binder interfaces as the delamination proceeded, which was proved by the numbers of water molecules, hydrogen bonds, and water clusters in interfacial regions. However, the number of water molecules and hydrogen bonds increased gradually even after most of the binder left the aggregate surface (snapshot 3), which was different from those at 10 m/s. This was because the pulling speed was so high that the water molecules were not able to fill the space in between in time. As shown by regions enclosed in the dashed ellipses, spaces with sparse water molecules were observed in snapshots 2 and 3. Eventually, water molecules filled these gaps.

**Figure 17:**
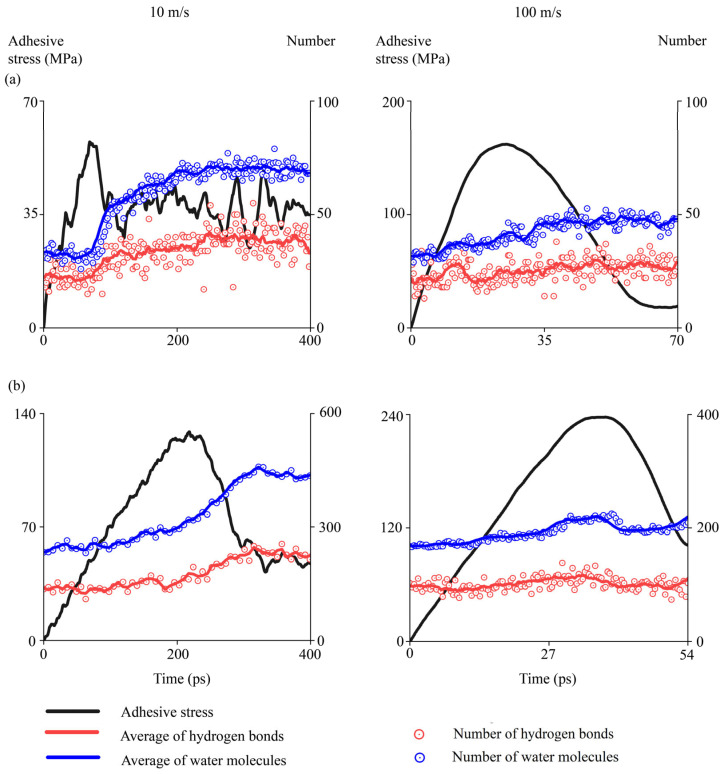
Evolutions of water molecules and hydrogen bonds when binders were delaminated from substrate at 10 m/s and 100 m/s. (**a**) The interface between AH 70 binder and silica. (**b**) The interface between AH 70 binder and epoxy.

Similarly, evolutions of water molecules and hydrogen bonds were examined for two more interfaces at 10 m/s and 100 m/s, i.e., the interface between AH 70 binder and silica, and the interface between AH 70 binder and epoxy (Figure 17). Results showed that water molecules propagated into aggregate–binder interfaces as delamination occurred. At a low speed of loading, water molecules first entered the interfaces gradually and then remained stable when most of the binder left the aggregates. At ~320 picoseconds, the number of water molecules and hydrogen bonds at the interface began to stabilize. At a high speed of loading, water molecules increased gradually during delamination, even after most of the binder left the aggregates, because it took an extra amount of time for water molecules to fill the gap in between. At ~80 picoseconds, the number of water molecules and hydrogen bonds at the interface became relatively stable. This contrast between distinctive loading rates underscored the critical role of water in altering the molecular interactions at asphalt–aggregate interfaces.

#### 4.2.9. Evolutions of Molecular Bonds During Interfacial Delamination

Different bonds existed at aggregate–binder interfaces in hydrated conditions, including bonds between aggregates and water, bonds between aggregates and binders, and bonds between asphalt binders and water. In this study, these bonds were calculated based on the cutoff radius of 10 Å. When the distance between two atoms was within 10 Å, it was considered that an atomic bond was formed. Evolutions of different atomic bonds were collected by tracking the number of bonds during the entire delamination process, during which the pulling simulations were performed well after sufficient relaxations described in prior sections. The sum of three types of bonds was used as the total number of bonds. Three representative interfaces were selected, i.e., the interface between AH 70 binder and silica, the interface between AH 70 and epoxy, and the interface between AH 70 binder with 4.5% SBS and epoxy. Evolutions of different bonds at low-speed or high-speed loadings, i.e., 20 and 200 m/s, were illustrated by showing the evolved the number of bonds during delamination between binders and aggregates in the hydrated conditions.

**Figure 18:**
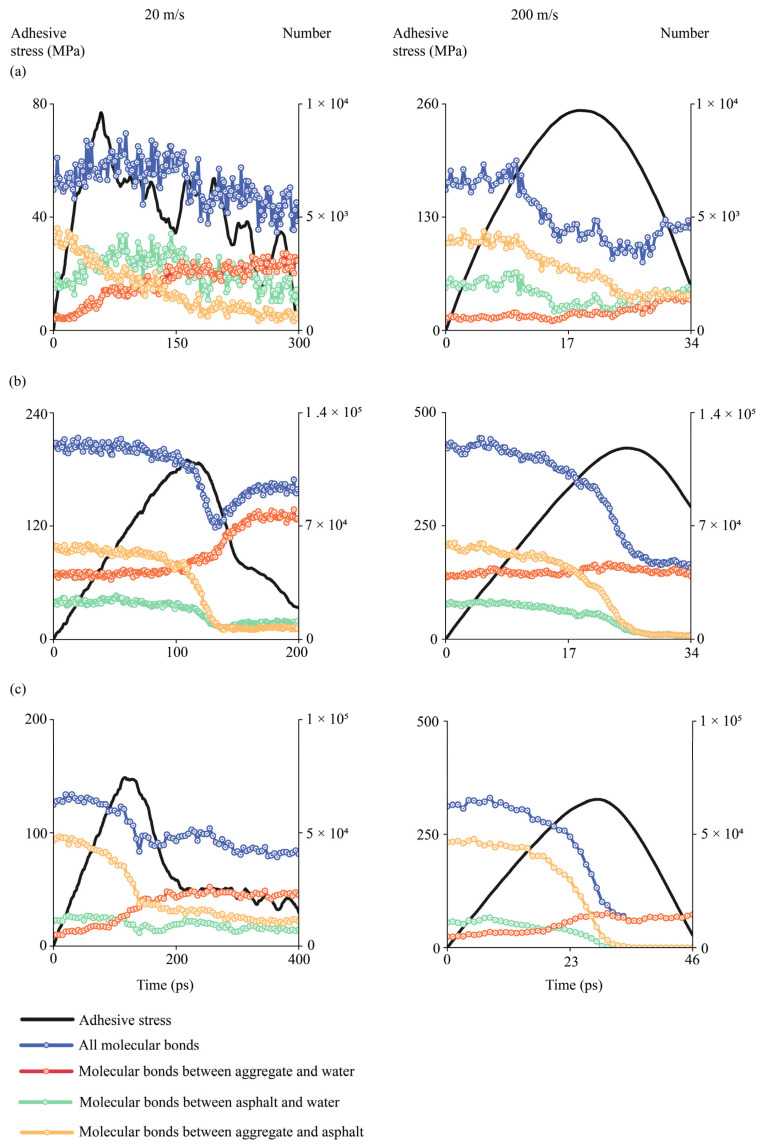
Evolutions of different bonds when binders were delaminated from the substrate at 20 m/s and 200 m/s. (**a**) The interface between AH 70 binder and silica. (**b**) The interface between AH 70 binder and epoxy. (**c**) The interface between AH 70 binder with 4.5% SBS and epoxy.

For the interface between the AH binder and silica (Figure 18a), it was shown that the number of bonds between aggregate and asphalt (yellow line) decreased slowly as the pulling force increased at the loading rate of 20 m/s. Upon reaching the peak force, the number of aggregate–asphalt bonds decreased substantially and eventually dropped to near zero. This was because most asphalt binders were separated from the interfacial region, leaving limited asphalt binders on aggregate surfaces. The number of bonds between asphalt and water (green line) increased in the beginning, then decreased slowly to a constant level. This was because at the low rate of loading, water molecules surrounding the asphalt binders slowly propagated into the asphalt–binder interface during the initial pulling. Then, a small amount of water molecules stayed around the residual of the asphalt binders after pulling. Similarly, the number of bonds between aggregate and water (red line) increased gradually and eventually remained at a high level. This was because water gradually invaded the interfacial aggregate surface during the pulling, and eventually covered the majority of the aggregate surface. The total number of bonds (blue line) increased slowly before peak pulling force and then decreased to a level lower than the initial number as the asphalt binders delaminated. The total number of bonds in the system was close to the number of bonds between aggregate and water at the later stage of pulling, meaning that major bonds at the aggregate–binder interface were formed by water and aggregate. Similar trends were observed for bond evolutions between the AH 70 binder and aggregate under the loading rate of 200 m/s. The difference was the evolution completed in a much shorter time period (~30 ps) than that at a low loading rate (~200 ps).

For the interface between AH 70 binder and epoxy at 20 m/s, similar trends were observed for the number of bonds between aggregate and water, the number of bonds between asphalt and water, and the number of bonds between aggregate and asphalt (Figure 18b). The total number of bonds after pulling were slightly lower than those before pulling. At the loading rate of 200 m/s, data analysis revealed that the number of bonds between asphalt and aggregate and between asphalt and water drops to zero after pulling. However, the number of bonds between aggregate and water remained almost at the same level during the pulling. This was because the pulling was so fast that it took a certain amount of time for water molecules to propagate into the interface. These findings were consistent with observations in prior sections. In the same vein, similar bond evolutions were observed for the interface between the AH binder with 4.5% SBS and epoxy (Figure 18c).

**Figure 19:**
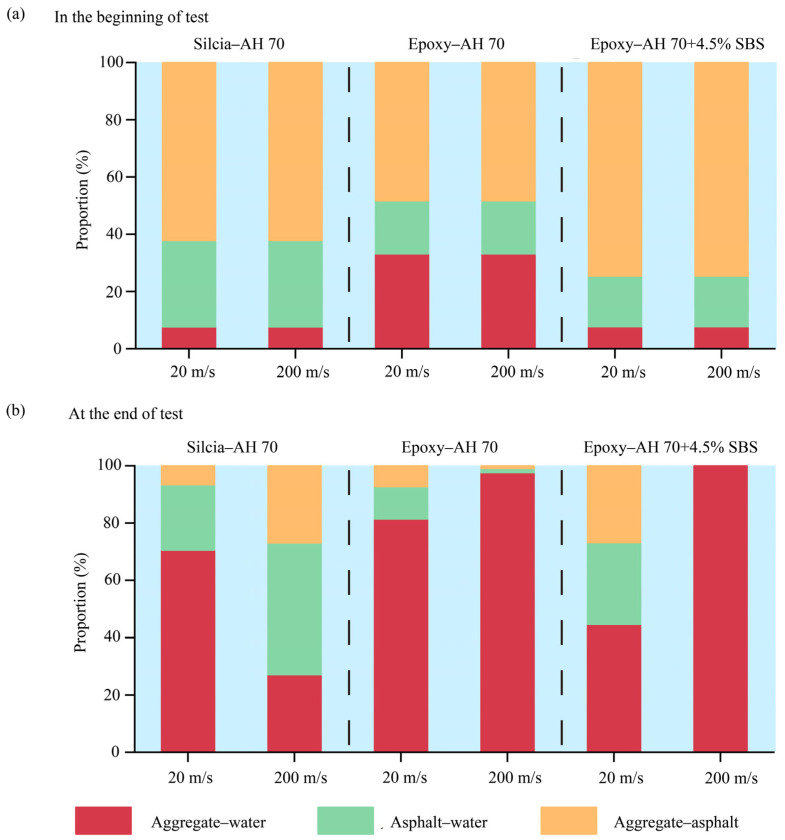
The proportions of different molecular bonds (**a**) in the beginning and (**b**) at the end of tests.

Fractions of molecular bonds at different interfaces in the beginning and at the end of tests are shown in Figure 19. It was shown that aggregate–asphalt bonds exhibited the largest fraction at the beginning of tests for both silica-based and epoxy-based aggregates. However, at the end of test, aggregate–water bonds occupied the most fraction of interfacial bonds. It was also shown that asphalt–water bonds and aggregate–water bonds exhibited certain fractions in silica-based interfaces.

## 5. Conclusions

The anticipated quantity of retired wind turbine blades in the near future will be a great challenge to the low-carbon utilization of wind energy. Meanwhile, the need for aggregates in road construction will be tremendous for the next few decades. In this study, the authors investigated the adhesion between glass fiber-reinforced composites and asphalt binder-reinforced composites at the molecular level to support the idea of using retired wind turbine blades to create high-performance asphalt mixtures.

To evaluate interfacial adhesion, molecular dynamics simulations were conducted between epoxy or minerals and various asphalt binders. Our findings revealed that adhesion between epoxy and asphalt binders was close to that between mineral aggregates and asphalt binders. The relationship between loading rates and adhesion was further investigated between epoxy and different SBS-modified asphalt binders. Distinctive regions were observed between adhesive forces and loading rates, i.e., high-speed, transitions, and low-speed regions. At low loading rates, a sufficient time for deformation and rupture of epoxy–binder interfaces resulted in lower adhesion, whereas simultaneous breakage of multiple bonds at epoxy–asphalt interfaces incurred large adhesion at high loading rates. Once the interfacial energy barrier was surmounted, epoxy–asphalt interfaces changed their behavior in response to the loading.

Water had a significant impact on the interfacial behavior between asphalt binders and aggregates. At low rates of loading, aggregate–binder adhesion was higher in the dry conditions than in hydrated conditions. However, aggregate–binder adhesion was lower in dry conditions than in hydrated conditions at high loading rates. This was because water provided extra delamination assistance to aggregate–binder interfaces, as they were separated gradually at low loading rates. However, at high rates of loading, water molecules were not able to propagate into aggregate–binder interfaces timely. The pulling of asphalt binders and the associated water molecules together during fast delamination resulted in higher adhesive forces.

At low loading rates, water propagated into aggregate–binder interfaces slowly in the beginning. The substantial increase in water molecules and hydrogen bonds occurred around peak force, after which numbers of water molecules remained stable. But at high rates of loading, the pulling speed was so fast that it took certain time for water molecules to propagate into the aggregate–binder interface, so the number of water molecules and hydrogen bonds increased consistently during pulling. At aggregate–binder interfaces, the number of bonds between water and aggregate increased during pulling, whereas the number of bonds between asphalt and water and the number of bonds between asphalt and water decreased to almost zero when pulling was completed. The number of bonds at aggregate–binder interfaces generally decreased during pulling.

Findings from this work show that good adhesive properties exist between asphalt binders and GFRP aggregates, providing essential support for the creation of asphalt mixtures with GFRP aggregates in practical applications [94]. Future work is needed to explore how salt and moisture affect the adhesion between binders and GFRP aggregates, along with the performance of asphalt mixture with GFRP aggregates. This research and implementation will pave the way for using recycled wind turbine blades on real roads.

## Figures and Tables

**Table 1:** Weight fractions of SARA components in different asphalt binders [46,47].

		Saturates	Aromatics	Resins	Asphaltenes
AH 70	wt%	4.3%	52.8%	31.6%	11.3%
PG 64-22	wt%	10.0%	38.3%	31.8%	19.9%

**Table 2:** The sizes of epoxy, alumina silica, and calcite substrates (Å).

	$L_{x}$ (Å)	$L_{y}$ (Å)	$L_{z}$ (Å)
Epoxy	85.05	85.05	25.00
Alumina	98.91	99.94	20.13
Silica	93.55	88.38	17.63
Calcite	115.66	119.76	19.77

**Table 3:** Surface morphology *R_h_* and energy *R_e_* of asphalt binders and aggregates.

Interfaces	Morphology RMS Rh (Å)		Energy Avg ± RMS Ea ± Re (kJ/mol)	
	Aggregate	Binder	Aggregate	Binder
Silica–AH 70 in dry condition	0.44	1.44	−2.62 ± 0.84	−2.23 ± 0.96
Epoxy–AH 70 in dry condition	3.30	1.98	−2.49 ± 1.16	−1.99 ± 0.97

## Data Availability

The original contributions presented in this study are included in the article/supplementary material. Further inquiries can be directed to the corresponding author.

## Supplementary Materials

The following supporting information can be downloaded at: https://www.mdpi.com/article/10.3390/ma18173936/s1, Table S1: Adhesive strength at different loading rates. Interfaces between AH 70 binders and different aggregate in dry and hydrated conditions; Table S2: Measured and simulated values for PG 64-22 with silica, and simulated values for binder with different aggregate; Figure S1: Brief schematic workflow.
