# Supplementary material for: Molecular Adhesion Between Asphalt and Glass Fiber-Reinforced Composites from Recycled Wind Turbine Blades in Dry and Hydrated Conditions
Source: Materials (Basel). 2025 Aug 22;18(17):3936. doi: 10.3390/ma18173936 (PMC12429557; doi:10.3390/ma18173936)
Supplement: Supplementary file 1 [file materials-18-03936-s001.zip › materials-3802968-supplementary.pdf]

Supplementary material

Table S1

Adhesive strength at different loading rates. Interfaces between AH 70 binders and different aggregate in dry and hydrated conditions.

v (m/s)		Unit surface energy (mJ/m²)			
		Dry		Hydrated	
		Epoxy	Silica	Epoxy	Silica
Control	2	148.61	100.36	99.46	41.07
	20	177.90	112.65	189.68	76.96
	200	259.10	207.26	344.63	162.11
2.5% SBS	2	99.80	140.65	69.38	104.88
	20	149.68	157.07	133.12	164.12
	200	247.50	300.44	300.19	386.01
4.5% SBS	2	121.35	86.10	72.75	41.67
	20	142.48	97.89	148.71	66.92
	200	267.51	238.47	327.11	290.36
6.5% SBS	2	189.83	103.07	77.00	38.59
	20	257.37	198.62	157.27	68.42
	200	414.24	268.92	404.46	231.78

Table S2

Measured and simulated values for PG 64-22 with silica, and simulated values for binder with different aggregate

Interface	γ (mJ/m²)
Silica - PG 64-22 (PPSFM)	85.05
Silica - PG 64-22 (MD)	59.52
Epoxy - PG 64-22 (MD)	65.08
Silica - AH 70 (MD)	68.14
Epoxy - AH 70 (MD)	105.56
Alumina - AH 70 (MD)	122.49

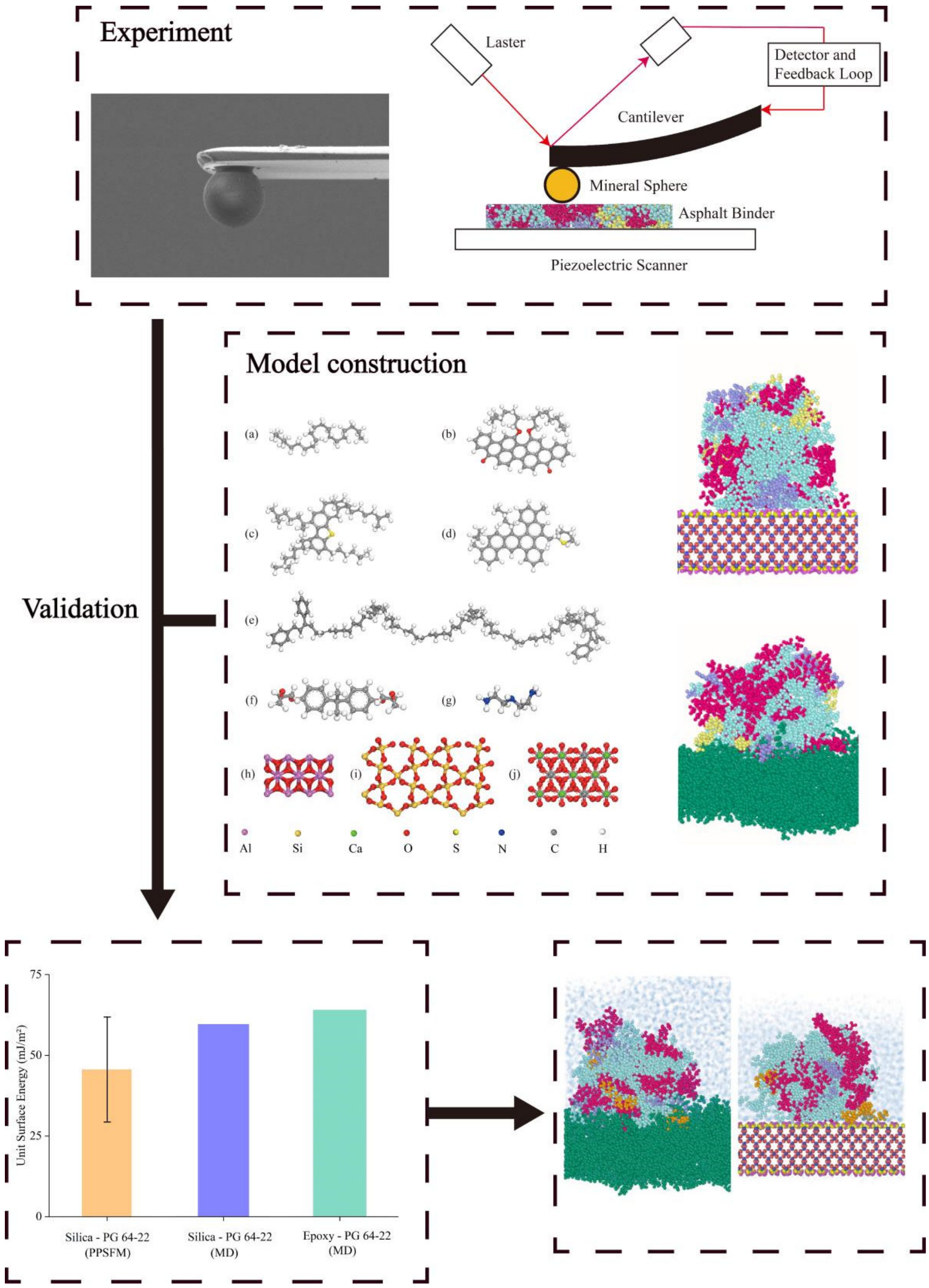

Figure S1. Brief schematic workflow
